# Bioactive selaginellins from *Selaginella tamariscina* (Beauv.) Spring

**DOI:** 10.3762/bjoc.8.217

**Published:** 2012-11-05

**Authors:** Chao Yang, Yutian Shao, Kang Li, Wujiong Xia

**Affiliations:** 1 State Key Lab of Urban Water Resource and Environment & the Academy of Fundamental and Interdisciplinary Sciences, Harbin Institute of Technology, Harbin 150080, PR China; 2 State Key Lab of Applied Organic Chemistry, Lanzhou University, Lanzhou 730000, PR China

**Keywords:** antioxidant, cytotoxicity, *Selaginella tamariscina*, selaginellin

## Abstract

A new selaginellin named selaginellin O (**1**), along with three other known selaginellins (**2**–**4**) were isolated from *Selaginella tamariscina* (Beauv.) Spring. On the basis of spectroscopic analysis, the structure of selaginellin O was demonstrated to be 4-[(4’-hydroxy-4-formyl-3-((4-hydroxyphenyl)ethynyl)biphenyl-2-yl)(4-hydroxyphenyl)methylene]cyclohexa-2,5-dien-1-one. Compound **1**, **2** and **3** exhibited appreciable cytotoxic activity against cultured HeLa cells (human cervical carcinoma cells), as well as significant antioxidant activity.

## Introduction

**Figure 1:**
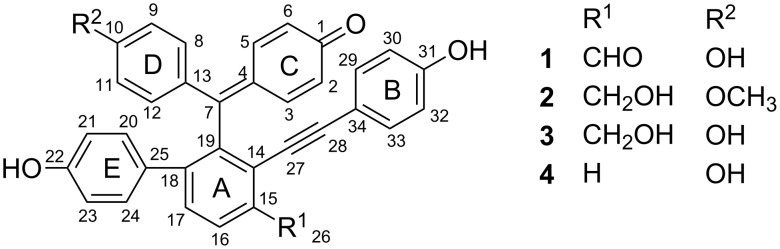
Structures of selaginellins from *S. tamariscina*.

There are about 700 species of the genus *Selaginella* (family selaginellacea) widely found in the world, with more than 50 species being found in China [1]. Twenty of them are widely used in Chinese folk medicine, most frequently employed for the treatment of cancer, cardiovascular problems, hepatitis, gastritis, hematuria, diabetes, and skin diseases [2]. *Selaginella tamariscina* (Beauv.) Spring is one of the two qualified species listed in Chinese Pharmacopoeia that has long been used as a traditional Chinese medicine for promoting blood circulation [3]. Phytochemical and pharmacological studies on genus *Selaginella* led to identifications of numerous bioactive compounds, including biflavonoids, alkaloids, and lignans, with broad biological activities, including antivirus, antifungal, antibacterial, cytotoxic, and anti-inflammatory properties [4–20]. In the past five years, more than 10 selaginellins (novel pigments with a unique *para*-quinone methide and alkynylphenol carbon skeleton) have been isolated from several *Selaginella* species in China [13–20]. Selaginellin derivatives have been hitherto found only in genus *Selaginella*. In the course of our phytochemical investigations on *Selaginella tamariscina* (Beauv.) Spring, four selaginellin derivatives (Figure 1), namely selaginellin M (**2**) [20], selaginellin (**3**) [13], selaginellin A (**4**) [14], and a new analogue selaginellin O (**1**), were isolated from the entire plant. Herein, we report the isolation and structural elucidation of these selaginellin derivatives, as well as the evaluation of their bioactivities.

## Results and Discussion

Selaginellin O (**1**), was obtained as a red powder, with the molecular formula C_34_H_22_O_5_, deduced from HRMS–ESI on the basis of the quasi-molecular ion peak at *m*/*z* 511.1543 [M + H]^+^ (calcd 511.1540). The IR spectrum indicated absorption bands for hydroxyl (3378 cm^−1^), formyl (2829, 2817, 1726 cm^−1^), alkynyl (2198 cm^−1^), unsaturated carboxyl (1680 cm^−1^) and aromatic ring (1570 and 1524 cm^−1^).

**Figure 2:**
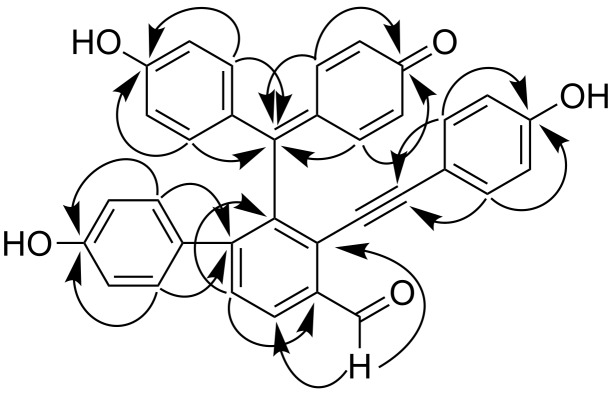
Key HMBC correlations of compound **1**.

The assignment of all ^1^H and ^13^C NMR data (shown in Table 1) was confirmed by 2D NMR techniques. The NMR spectra of **1** showed the typical signals of a formyl group (δ_H_ 10.73 and δ_C_ 190.9), an alkynyl band (δ_C_ 82.4, 101.0), three phenolic hydroxyl (δ_H_ 8.94 and 8.59), and five aromatic rings, including one AB-spin system (δ_H_ 7.52 and 8.05, each 1H, d, *J* = 8.0 Hz) for the *ortho*-tetrasubstituted A-ring, three AA’BB’ systems (δ_H_ 7.22 and 6.78, each 2H, d, *J* = 8.4 Hz), (δ_H_ 6.87 and 6.71, each 2H, d, *J* = 8.4 Hz) and (δ_H_ 6.96 and 6.70, each 2H, d, *J* = 8.4 Hz) for the respective *para*-substituted B-, D- and E-ring, and one ABMN system (δ_H_ 7.61, 7.42, 6.41 and 6.35, each 1H, d, *J* = 10.0 Hz) for the C-ring. The above structural features suggested **1** was a selaginellin with a formyl group. Key evidence for the structure of **1** obtained from the HMBC experiment further confirmed this suggestion (Figure 2). The HMBC correlations H16/C-26 and H-26/C-15 concluded the substitution of the formyl group at C-15 of the A-ring. The alkynyl group was connected to the B-ring based on correlations between H-29,33 and C-28. The linkage between the C-ring and the B-ring was located at C-7 demonstrated by the HMBC cross-peaks of H-3,5/C-7 and H-8,12/C-7. The E-ring was connected to the A-ring at C-18 due to HMBC correlations H-20,24/C-18 and H-17/C-25. Hence, C-19 in the A-ring was the position left for C-7. Consequently, the structure of compound **1** was characterized as 4-[(4’-hydroxy-4-formyl-3-((4-hydroxyphenyl)ethynyl)biphenyl-2-yl)(4-hydroxyphenyl)methylene]cyclohexa-2,5-dien-1-one, named as selaginellin O.

**Table 1:** ^1^H and ^13^C NMR data and key HMBC correlations for compound **1**.^a^

Position	δ_H_	δ_C_	(DEPT)	HMBC (H→C)

1	–	185.6	(qC)	–
2	6.35 d (10.0)	128.4	(CH)	C-4
3	7.42 d (9.6)	139.2	(CH)	C-1,7
4	–	131.4	(qC)	–
5	7.61 d (9.6)	138.1	(CH)	C-1,7
6	6.41 d (10.0)	128.5	(CH)	C-4
7	–	156.3	(qC)	–
8	6.87 d (8.4)	133.0	(CH)	C-7,10
9	6.71 d (8.4)	114.9	(CH)	C-13
10	–	158.8	(qC)	–
11	6.71 d (8.4)	114.9	(CH)	C-13
12	6.87 d (8.4)	133.0	(CH)	C-7,10
13	–	131.4	(qC)	–
14	–	127.7	(qC)	–
15	–	134.2	(qC)	–
16	8.05 d (8.0)	127.5	(CH)	C-14,18,26
17	7.52 d (8.0)	130.1	(CH)	C-15,19,25
18	–	148.1	(qC)	–
19	–	142.3	(qC)	–
20	6.96 d (8.4)	129.8	(CH)	C-18, 22
21	6.70 d (8.4)	114.9	(CH)	C-25
22	–	157.4	(qC)	–
23	6.70 d (8.4)	114.9	(CH)	C-25
24	6.96 d (8.4)	129.8	(CH)	C-18, 22
25	–	130.8	(qC)	–
26	10.73 s	190.9	(CH)	C-14, 16
27	–	82.4	(qC)	–
28	–	101.0	(qC)	–
29	7.22 d (8.4)	133.5	(CH)	C-28, 31
30	6.78 d (8.4)	115.6	(CH)	C-34
31	–	158.7	(qC)	–
32	6.78 d (8.4)	115.6	(CH)	C-34
33	7.22 d (8.4)	133.5	(CH)	C-28, 31
34	–	112.5	(qC)	–
10-OH	8.94 (*br*)^b^			C-9, 11
22-OH	8.59 (*br*)			C-21, 23
31-OH	8.94 (*br*)^b^			C-30, 32

^a^400 MHz, acetone-*d*_6_, δ in parts per million, *J* in hertz. ^b^Overlapping signals.

The chemical structures of selaginellin M (**2**), selaginellin (**3**) and selaginellin A (**4**) were identified by spectral analysis by 1D and 2D NMR spectroscopy and high-resolution MS. Their ^1^H and ^13^C NMR data were assigned in Table 2.

**Table 2:** ^1^H and ^13^C NMR data for compound **2**, **3** and **4**.^a^

	**2**		**3**		**4**	

Position	δ_H_	δ_C_	δ_H_	δ_C_	δ_H_	δ_C_

1	–	185.7	–	173.1	–	185.9
2	6.32 dd (2.0, 10.0)	128.1	6.54 d (8.8)	121.4	6.35 m	128.2
3	7.34 dd (2.4, 10.0)	139.5	7.16 d (9.2)	136.5	7.48 d (8.0)	139.5
4	–	129.7	–	130.2	–	128.4
5	7.52 dd (2.4, 10.0)	138.1	7.16 d (9.2)	136.5	7.59 d (8.0)	138.0
6	6.36 dd (2.0, 10.0)	130.2	6.54 d (8.8)	121.4	6.35 m	128.2
7	–	158.1	–	160.1	–	158.0
8	6.88 d (9.2)	132.7	7.16 d (9.2)	136.5	6.75 d (8.8)	133.0
9	6.66 d (8.8)	113.2	6.54 d (8.8)	121.4	6.64 m	114.7
10	–	160.8	–	173.1	–	158.8
11	6.66 d (8.8)	113.2	6.54 d (8.8)	121.4	6.64 m	114.7
12	6.88 d (9.2)	132.7	7.16 d (9.2)	136.5	6.75 d (8.8)	133.0
13	–	131.7	–	130.2	–	133.5
14	–	121.5	–	121.7	–	124.6
15	–	142.7	–	142.5	7.67 d (8.0)	130.2
16	7.79 d (8.0)	126.8	7.79 d (8.0)	127.0	7.59 t (7.6)	129.1
17	7.38 d (8.0)	129.7	7.37 d (8.0)	129.7	7.48 d (8.0)	130.0
18	–	140.9	–	141.3	–	143.0
19	–	141.0	–	140.9	–	141.1
20	6.91 d (8.4)	130.2	6.87 d (8.4)	129.9	6.89 d (8.4)	129.8
21	6.80 d (9.2)	114.7	6.65 d (8.4)	114.8	6.64 m	114.7
22	–	156.7	–	156.8	–	156.8
23	6.80 d (9.2)	114.7	6.65 d (8.4)	114.8	6.64 m	114.7
24	6.91 d (8.4)	130.2	6.87 d (8.4)	129.9	6.89 d (8.4)	129.8
25	–	131.7	–	131.6	–	131.7
26	5.01 s	62.2	5.02 s	62.2		
27	–	83.8	–	83.8	–	86.6
28	–	98.8	–	99.0	–	93.5
29	7.11 d (8.4)	133.0	7.09 d (8.4)	133.0	7.09 d (8.8)	133.1
30	6.76 d (8.8)	115.5	6.75 d (8.4)	115.6	6.74 d (8.4)	115.5
31	–	157.8	–	158.4	–	158.0
32	6.76 d (8.8)	115.5	6.75 d (8.4)	115.6	6.74 d (8.4)	115.5
33	7.11 d (8.4)	133.0	7.09 d (8.4)	133.0	7.09 d (8.8)	133.1
34	–	113.6	–	113.3	–	113.6
-OMe	3.79 s	54.8				

^a^400 MHz, acetone-*d*_6_, δ in parts per million, *J* in hertz.

Several species of the genus *Selaginella* have long been used in traditional medicine as anticancer agents, but only limited literature information on the cytotoxic activity of their constituents is available, which encourages us to investigate the cytotoxic effect of the seleginellins [4–5,7]. The cytotoxic activities of the three selaginellins: selaginellin O (**1**), selaginellin M (**2**) and selaginellin (**3**) were evaluated by using human cervical carcinoma (HeLa) cells. It is noticeable that all of these selaginellins exhibited appreciable cytotoxic activity (Supporting Information File 1; Table S1). Selaginellin O (**1**), the new selaginellin with a formyl group, showed the highest inhibitory activity, with an IC_50_ value of 26.4 μM. Consistent with earlier literature [20], the considerable inhibition of the expression of HeLa cells was observed for the known compounds selaginellin M (**2**) and selaginellin (**3**), with IC_50_ equal to 28.5 and 33.1 μM, respectively.

Over the past few decades, considerable biochemical, physiological and pharmacological evidence has accumulated to support the hypothesis that free-radical-mediated oxidative processes are implicated in various human diseases. The role of free radicals in ageing, in cancer, and in cardiovascular, neurodegenerative and other diseases is more and more widely accepted [21–22]. Antioxidants are attracting increasing scientific and clinical attention.

Natural phenolic compounds (flavonoids, lignans, phenolic acids, tocopherols, polyphenols and tannins) are the main class of antioxidants and are known to reduce the rate of oxidation by H-transfer (from their phenol groups) to the radicals [23]. There are multiple phenol groups on the large conjugated aromatic skeleton of selaginallins, and this potential antioxidant structural feature motivated the study on their antioxidant activity. Accordingly, the antioxidant capacity of selaginellin O (**1**), selaginellin M (**2**) and selaginellin (**3**) were estimated with the widely used ABTS radical-scavenging assay and FRAP assay. In both assays, all the selaginellins tested displayed significant antioxidant activity, with more potent antioxidant capacity than the reference compound Trolox (Supporting Information File 1; Table S2). Likewise, the new compound, selaginellin O (**1**), exhibited the highest antioxidant activity, and a general increase in antioxidant activity was observed compared with its reduced form selaginellin (**3**). This may be correlated with the electron-accepting and delocalization effects of the formyl group, which favor the ionization of ArOH to the phenoxide anion ArO^−^, benefitting from both H-transfer and stabilization of the important mediator ArO^−^. The above two main factors of the antioxidant contribute to the potent antioxidant properties of selaginallin O.

The current pharmacological evidence shows that antioxidant treatment may significantly inhibit atherosclerosis, which indicates that selaginallins may be partly related to the herbal use of *S. tamariscina* for promoting blood circulation and eliminating blood stasis [24].

Despite its preliminary character, this study is the first to report the antioxidant activity of the selaginellins. These promising biological results and the structural specificity of the selaginellins stimulated us to search for more potent and selective selaginellin analogues and their biogenetic precursors. Additional controlled studies are needed to investigate the efficacy and safety of selaginellins as antioxidant and anticancer agents.

## Experimental

**General experimental procedures.** IR spectra were measured on a Perkin Elmer Spectrum 100 FT-IR Spectrometer. All NMR spectra data were recorded on a Bruker 400 MHz AVANCE III FT-NMR spectrometer operating at 400 MHz for ^1^H and 100 MHz for ^13^C NMR by using TMS as the internal standard. HRMS–ESI spectrum was obtained on an Agilent 1200-6520 QTOF. UV spectra were recorded on a Perkin Elmer LAMBDA 750 UV/Vis/NIR spectrophotometer. The melting point was measured by Büchi B-540 Melting Point Apparatus. Optical rotations were measured with a WZZ-3 automatic Polarimeter. Column chromatography was performed over silica gel (200–300 mesh). Thin-layer chromatography (TLC) was conducted on precoated silica gel plates GF_254_ (Qingdao Marine Chemical Factory). 3-(4,5-Dimethylthiazol-2-yl)-2,5-diphenyltetrazolium bromide (MTT), cisplatin, and 2,4,6-tri(2-pyridyl)-1,3,5-triazine (TPTZ) were from Aladdin Reagent Co. Ltd. 2,2’-azinobis(3-ethylbenzothiazoline-6-sulfonic acid) diammonium salt (ABTS) and 6-hydroxy-2,5,7,8-tetramethylchroman-2-carboxylic acid (Trolox) were obtained from Sigma.

**Plant material.** The herb of *Selaginella tamariscina* was purchased from Tongtai Medicine, Harbin Co. Ltd., China. The material was authenticated by Prof. Zhenyue Wang, Heilongjiang University of Chinese Medicine. A voucher specimen (No. 200705JB) was deposited in the Lab of Applied Organic Chemistry, Harbin Institute of Technology.

**Extraction and isolation.** Dried whole herb of *S. tamariscina* (5.0 kg) was pulverized and extracted with methanol (3 × 5.0 L) at room temperature. The combined methanolic extract was concentrated in vacuum giving a dark residue (605 g), which was partitioned into five fractions, petroleum ether (80 g), Et_2_O (41 g), EtOAc (112 g), Me_2_CO (96 g) and MeOH (221 g), by silica-gel column chromatography. The ethyl acetate fraction (112 g) was chromatographed on silica gel (chloroform/methanol 1:0→0:1) to afford fractions 1–20. Fraction 15 (600 mg) was resubjected to silica gel CC with gradient petrol ether/acetone (10:1→0:1) to give amentoflavone (328.4 mg) and some red powder, which was then further purified on PTLC (petrol ether/acetone 4:1) to afford selaginellin M (**2**) (1.83 mg) and selaginellin O (**1**) (2.37 mg). Selaginellin (**3**) (83.0 mg) and selaginellin A (**4**) (0.7 mg) were obtained from fraction 16 (152 mg) by CC (silica gel; petrol ether/ethyl acetate 10:1, 5:1, 2:1, 1:1 and 1:5), followed by PTLC (chloroform/methanol 15:1).

**Selaginellin O (1)**: Red powder; UV (MeOH) λ_max_ (log ε): 298 (3.15), 415 (1.8) nm; IR (NaCl) ν_max_: 3378, 2198, 2829, 2817, 1726, 1680, 1567 and 1524 cm^−1^; ^1^H and ^13^C NMR (DEPT) data were shown in Table 1; HRMS–ESI (*m*/*z*): [M + H]^+^ calcd for C_34_H_23_O_5_^+^, 511.1540; found, 511.1543.

**Selaginellin M (2)**: Red powder; UV (MeOH) λ_max_ (log ε): 297 (3.15), 431 (1.05); IR (NaCl) ν_max_: 3420, 2196, 1708, 1545 and 1535 cm^−1^; ^1^H and ^13^C NMR (DEPT) data were shown in Table 2; HRMS–ESI (*m*/*z*): [M + H]^+^ calcd for C_35_H_27_O_5_^+^, 527.1853; found, 527.1868.

**Selaginellin (3)**: Red crystals (MeOH); IR (NaCl) ν_max_: 3387, 2195, 1689, 1595 and 1531 cm^−1^; ^1^H and ^13^C NMR data were shown in Table 2; HRMS–ESI (*m*/*z*): [M + H]^+^ calcd for C_34_H_25_O_5_^+^, 513.1697; found, 513.1702.

**Selaginellin A (4)**: Red powder; IR (NaCl) ν_max_: 3404, 2205, 1656, 1575 and 1531 cm^−1^; ^1^H and ^13^C NMR data were shown in Table 2; HRMS–ESI (*m*/*z*): [M + H]^+^ calcd for C_33_H_23_O_4_^+^, 483.1591; found, 483.1587.

**Cytotoxicity assay.** Due to insufficient material, selaginellin O (**1**), selaginellin M (**2**) and selaginellin (**3**), were evaluated for their cytotoxic activity against cultured human cervical carcinoma HeLa cells by using the MTT [3-(4,5-dimethylthiazol-2-yl)-2,5-diphenyltetrazolium bromide] colorimetric method [25]. The anticancer agent cisplatin was used as a positive control. The cytotoxicity data were expressed as IC_50_ (half inhibition concentration) values.

**ABTS radical scavenging assay.** The assay was performed according to the established protocol [26]. The ABTS^•+^ radical was generated by mixing 7 mM aqueous ABTS solution with 2.45 mM potassium persulfate solution (final concentration) followed by incubation in the dark at room temperature for 16 h. The resultant ABTS^•+^ solution was diluted with ethanol to give an absorbance of 0.70 ± 0.02 at 734 nm and equilibrated at 30 °C. After the addition of 2.85 mL of diluted ABTS^•+^ solution to 0.15 mL of different concentrations of samples in ethanol, the absorbance reading was taken at 30 °C, exactly 6 min after the initial mixing. The percentage inhibition of absorbance at 734 nm was calculated and plotted as a function of the concentration of samples and of Trolox as standard. The antioxidant activities are expressed as the IC_50_ value.

**FRAP (Ferric reducing antioxidant power) assay.** This assay was carried out following the procedure described previously with modifications [27]. FRAP reagent was prepared fresh by mixing 10 mM TPTZ, 20 mM FeCl_3_ and 300 mM acetate buffer (pH 3.6) in a 1:1:10 (v/v/v) ratio and warmed to 37 °C before use. A 0.2 mL amount of the sample including Trolox as a reference compound in methanol was added to 1.8 mL freshly prepared FRAP reagent and then incubated at 37 °C for 8 min. Absorbance of the resulting Fe^II^-TPTZ (ferrous-tripyridyltriazine) complex was measured at 595 nm. Antioxidant power was expressed as micromolar Fe^II^-TPTZ equivalents, calculated from a calibration curve prepared with various concentrations of FeSO_4_.

## Supporting Information

File 1 Spectroscopic data and other relevant information for compounds **1**–**4**.
