# Supplementary material for: Bioactive selaginellins from Selaginella tamariscina (Beauv.) Spring
Source: Beilstein J Org Chem. 2012 Nov 5;8:1884–9. doi: 10.3762/bjoc.8.217 (PMC3511026; doi:10.3762/bjoc.8.217)
Supplement: File 1 — Spectroscopic data and other relevant information for compounds 1–4. [file Beilstein_J_Org_Chem-08-1884-s001.pdf]

Supporting Information

for

Bioactive selaginellins from *Selaginella tamariscina* (Beauv.) Spring

Chao Yang *[1,2], Yutian Shao [1], Kang Li [1] and Wujiong Xia [1]

Address: [1] State Key Lab of Urban Water Resource and Environment & the Academy of Fundamental and Interdisciplinary Sciences, Harbin Institute of Technology, Harbin 150080, PR China and [2] State Key Lab of Applied Organic Chemistry, Lanzhou University, Lanzhou 730000, PR China

Email: Chao Yang* - xyyang@hit.edu.cn

* Corresponding author

Spectroscopic data and other relevant information for compounds 1–4.

Table of Contents

Figure S1 ^{1}H NMR (400 MHz, CD_3COCD_3) of compound **1** S3

Figure S2 ^{13}C NMR (100 MHz, CD_3COCD_3) of compound **1** S3

Figure S3 ^{1}H-^{1}H COSY of compound **1** S4

Figure S4 HMQC of compound **1** S5

Figure S5 HMBC of compound **1** S6

Figure S6-S10 ^{1}H, ^{13}C and 2D NMR spectra of compound **2** S7–S9

Figure S11-S15 ^{1}H, ^{13}C and 2D NMR spectra of compound **3** S10–S12

Figure S16-S20 ^{1}H, ^{13}C and 2D NMR spectra of compound **4** S13–S15

Table S1 Cytotoxic activity data for compounds **1**–**3** S16

Table S2 Antioxidant activity data for compounds **1**–**3** S16

Figure S1: ^{1}H NMR (400 MHz, CD_3COCD_3) of compound **1**

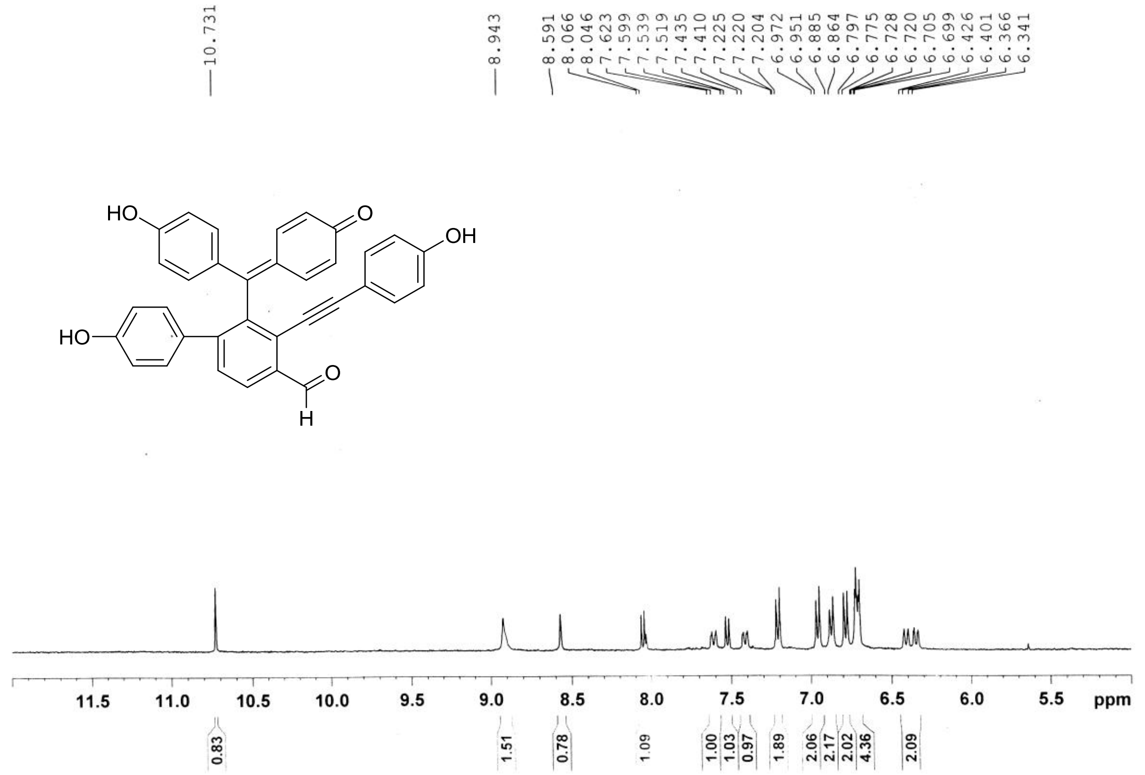

Figure S2: ^{13}C NMR (100 MHz, CD_3COCD_3) of compound **1**

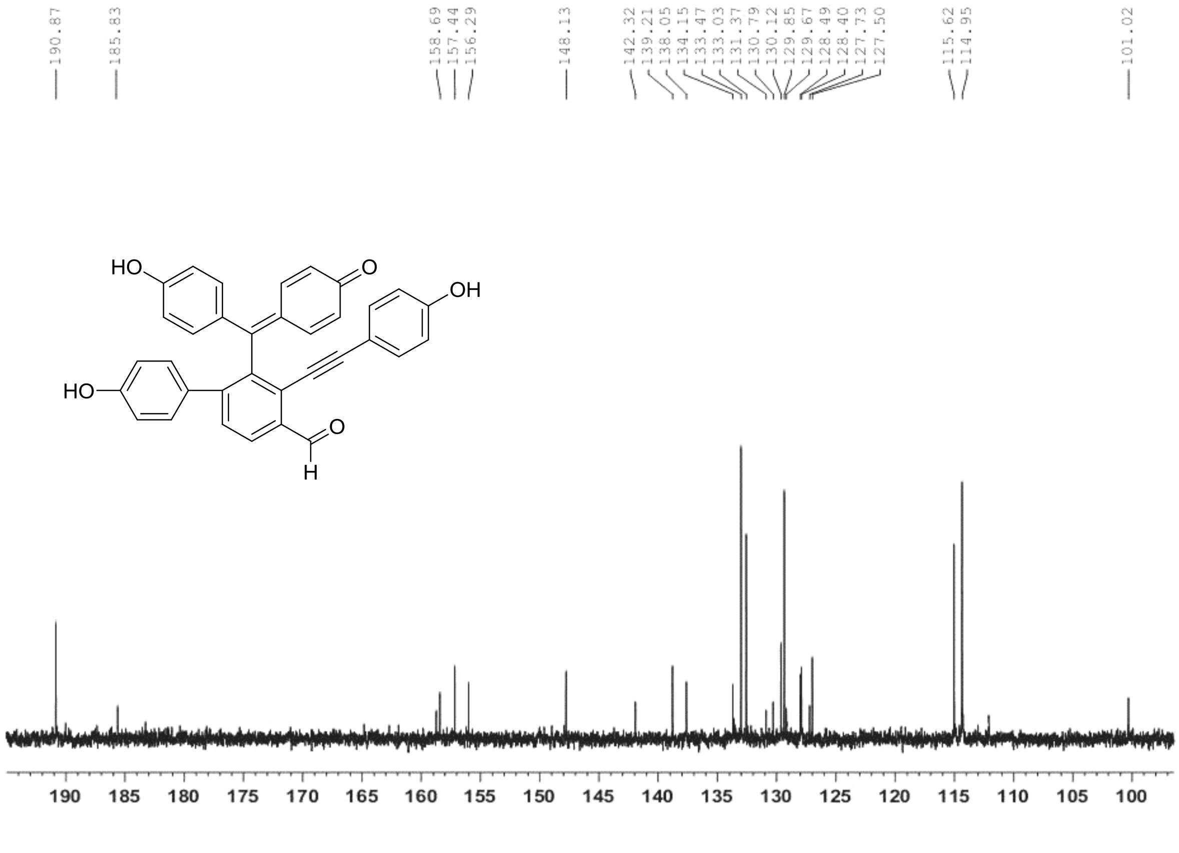

Figure S3: ^{1}H-^{1}H COSY of compound **1**

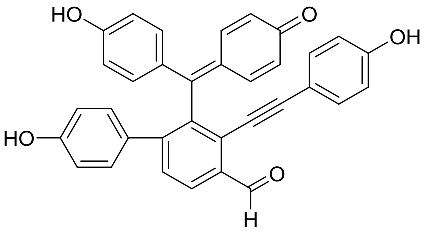

Selaginellin O

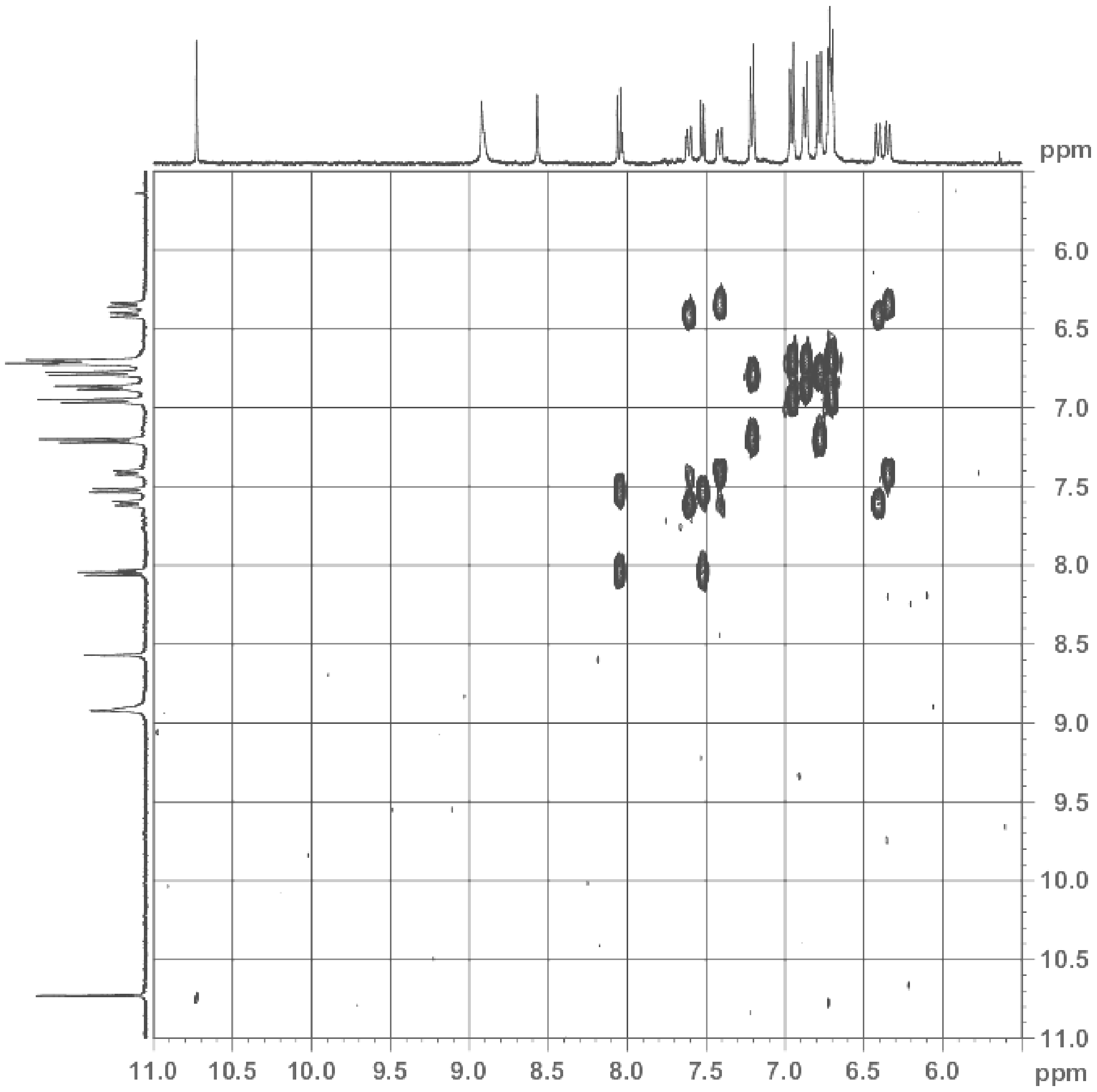

Figure S4: HMQC of compound **1**

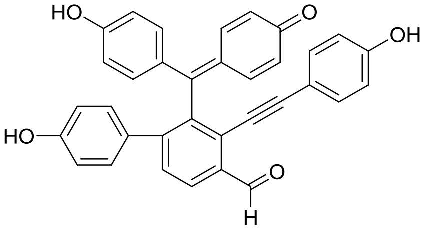

Selaginellin O

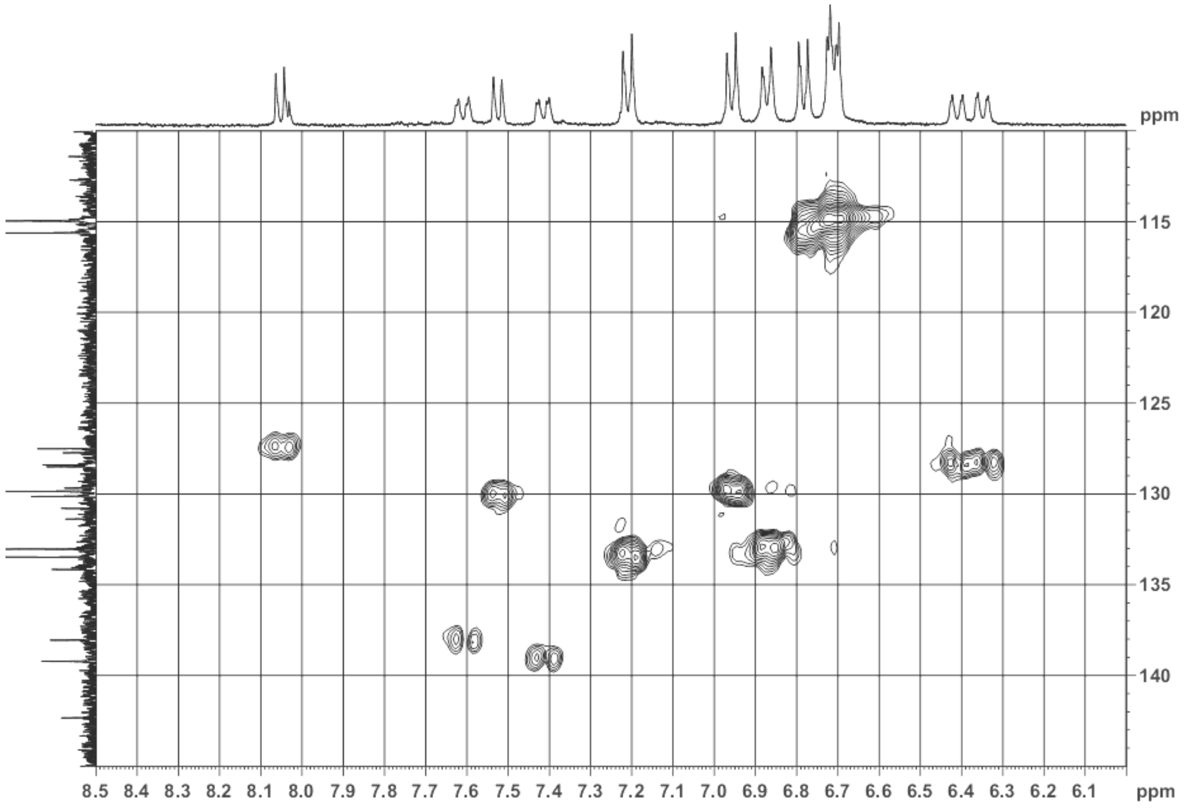

Figure S5: HMBC of compound **1**

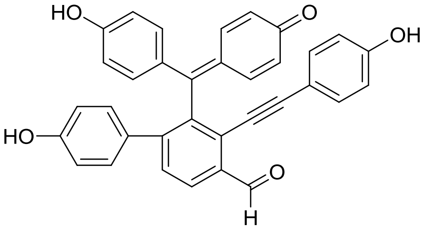

Selaginellin O

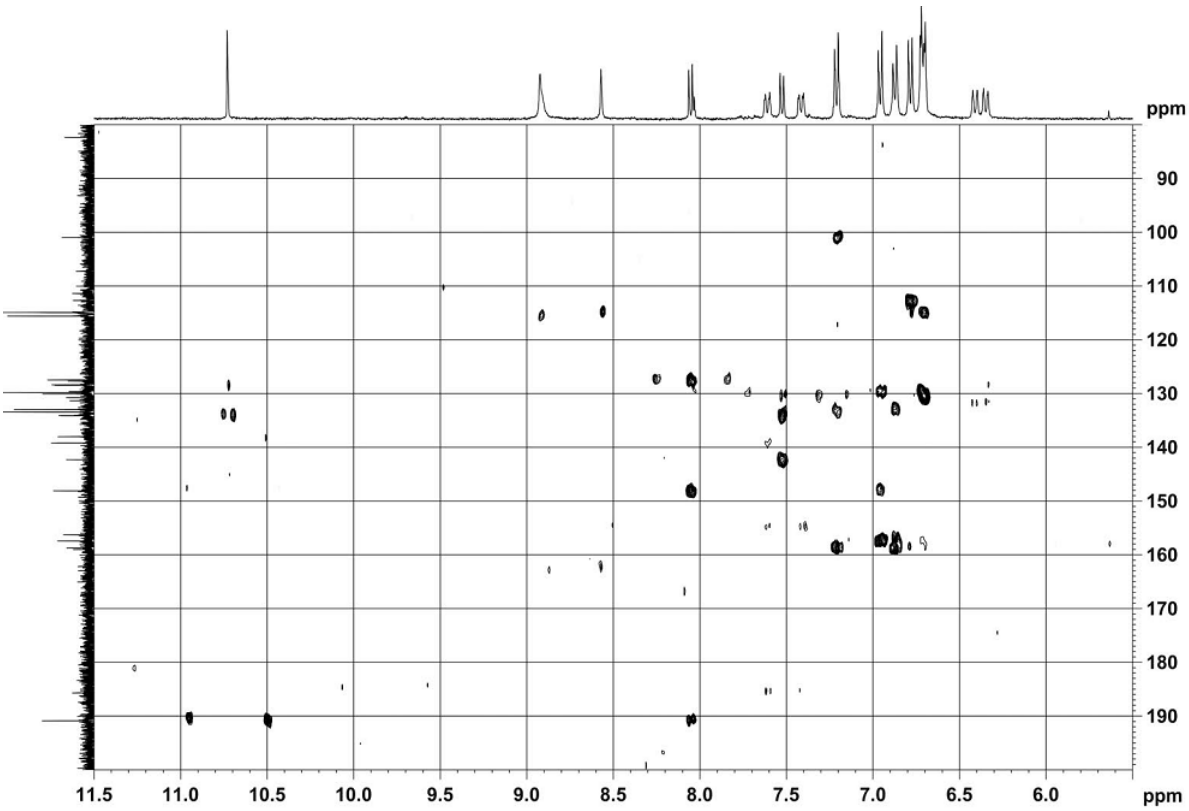

Figure S6: ^{1}H NMR (400 MHz, CD_3COCD_3) of compound **2**

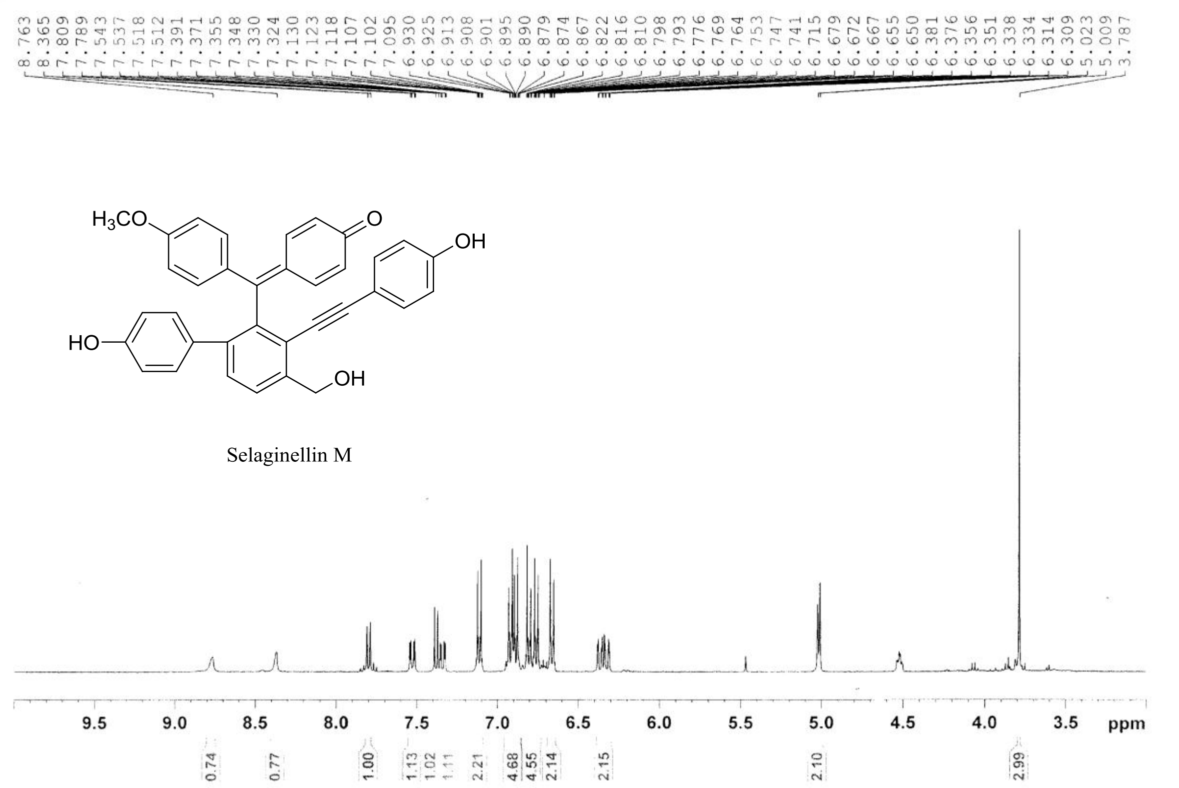

Figure S7: ^{13}C NMR (100 MHz, CD_3COCD_3) of compound **2**

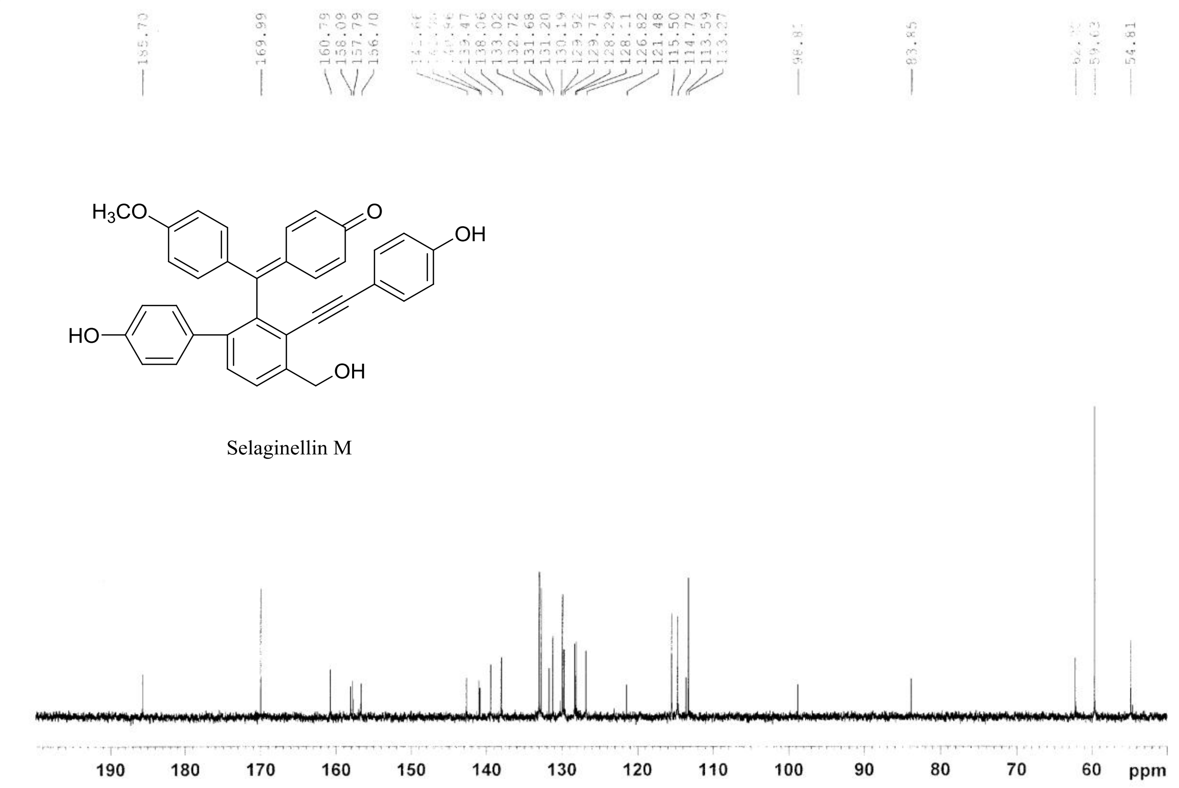

Figure S8: ^{1}H-^{1}H COSY of compound **2**

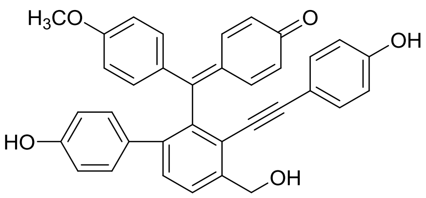

Selaginellin M

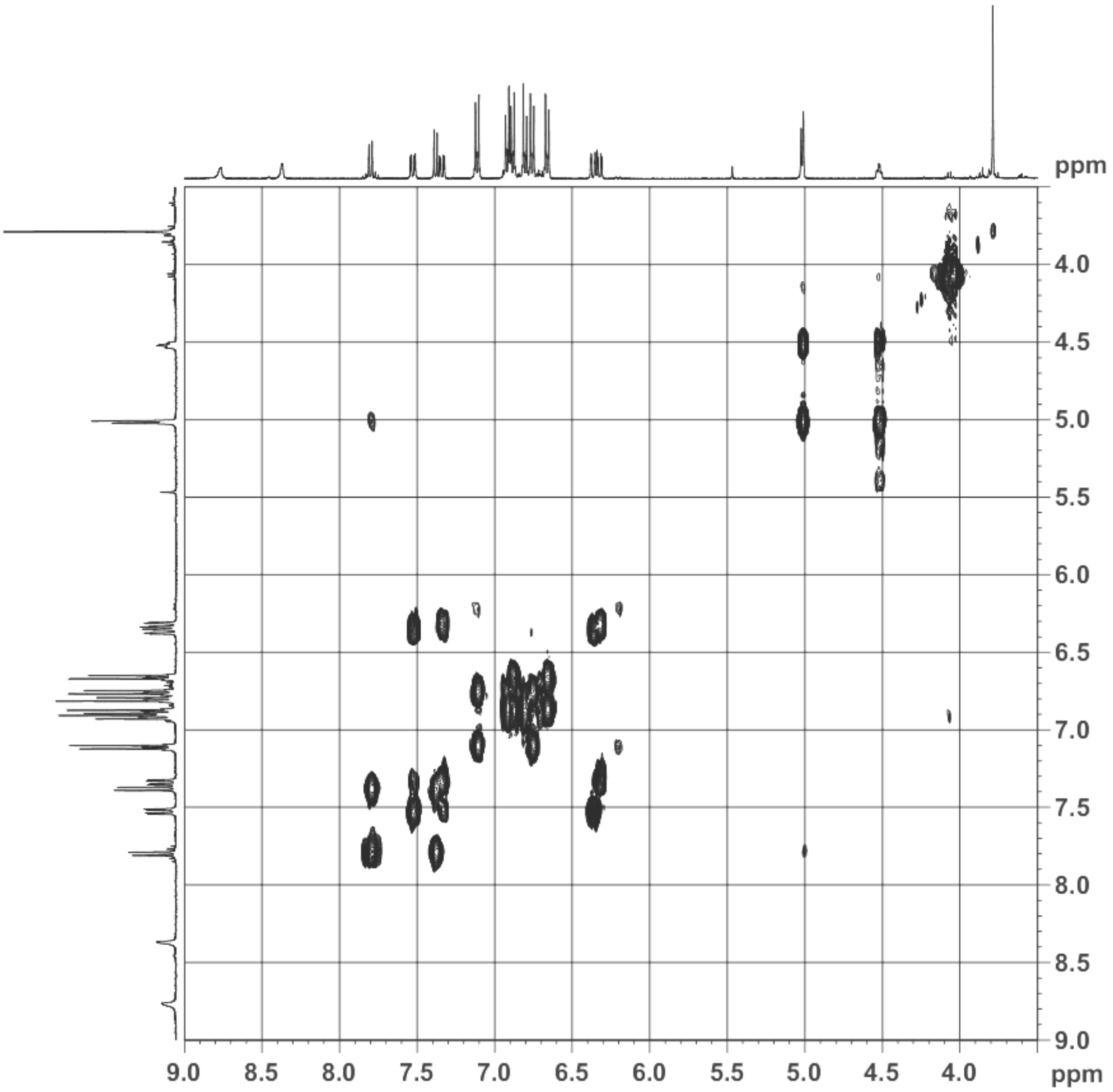

Figure S9: HMQC of compound **2**

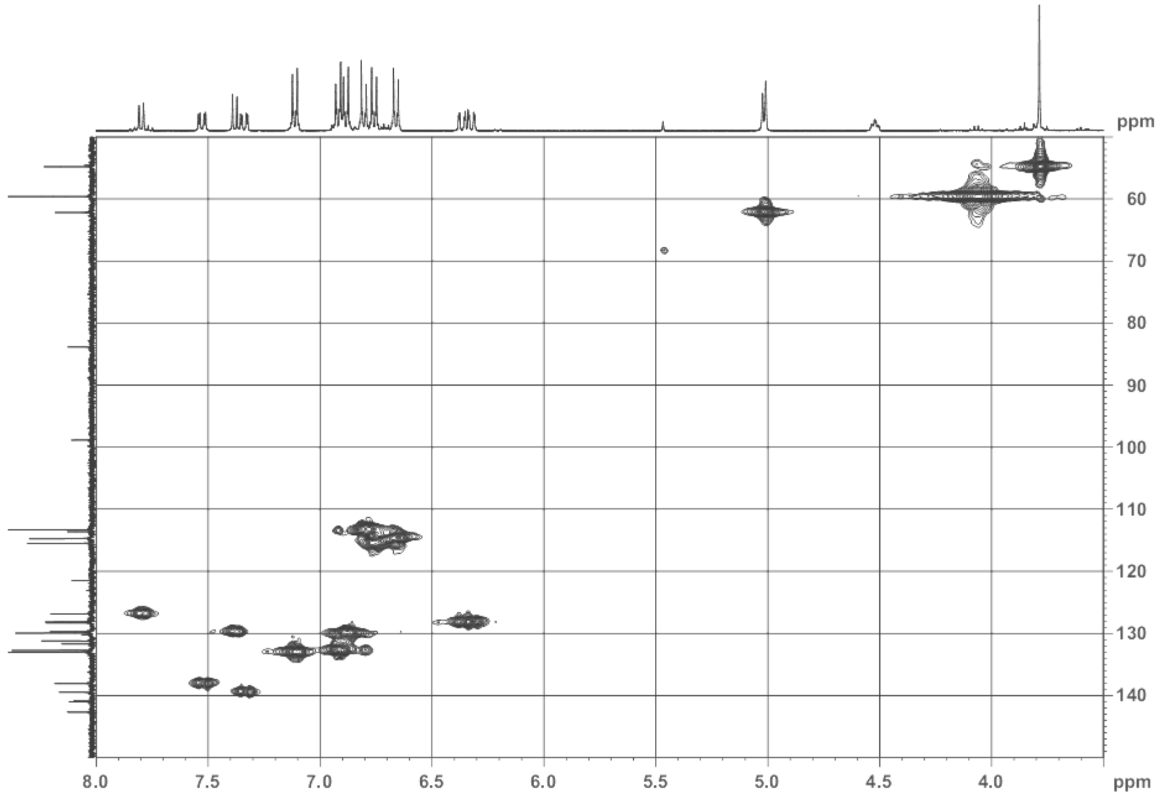

Figure S10: HMBC of compound **2**

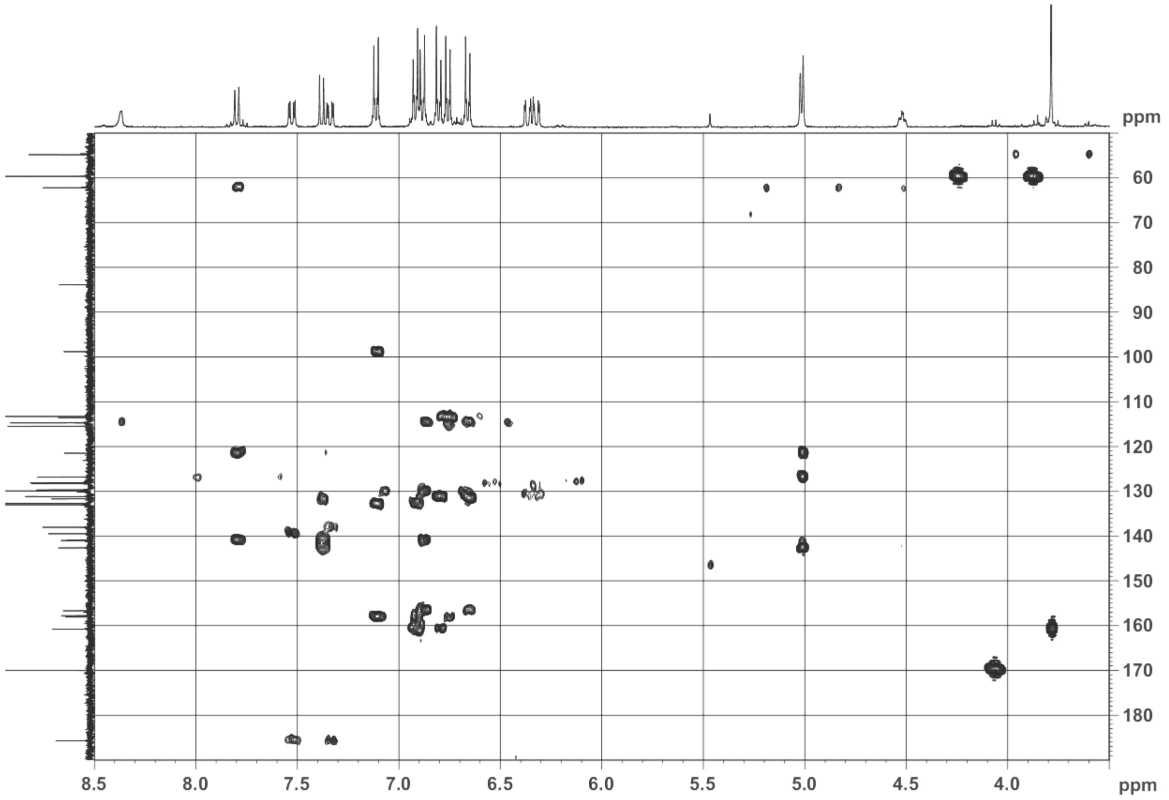

Figure S11: ^{1}H NMR (400 MHz, CD_3COCD_3) of compound **3**

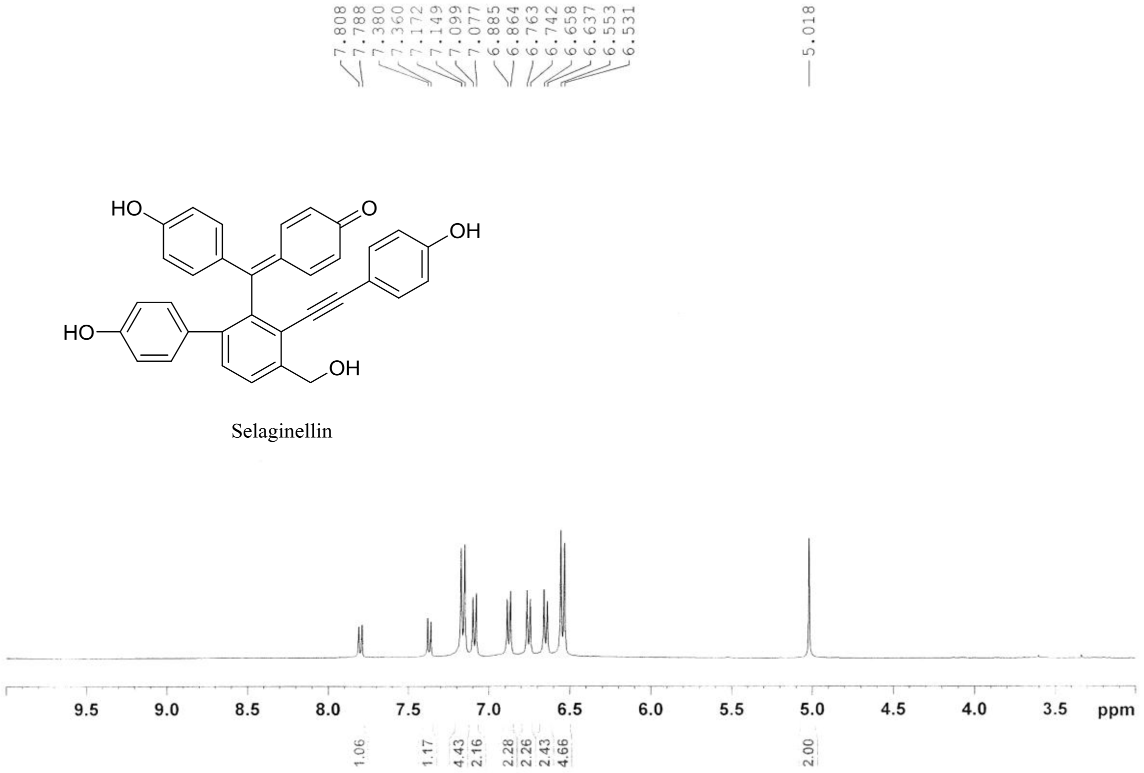

Figure S12: ^{13}C NMR (100 MHz, CD_3COCD_3) of compound **3**

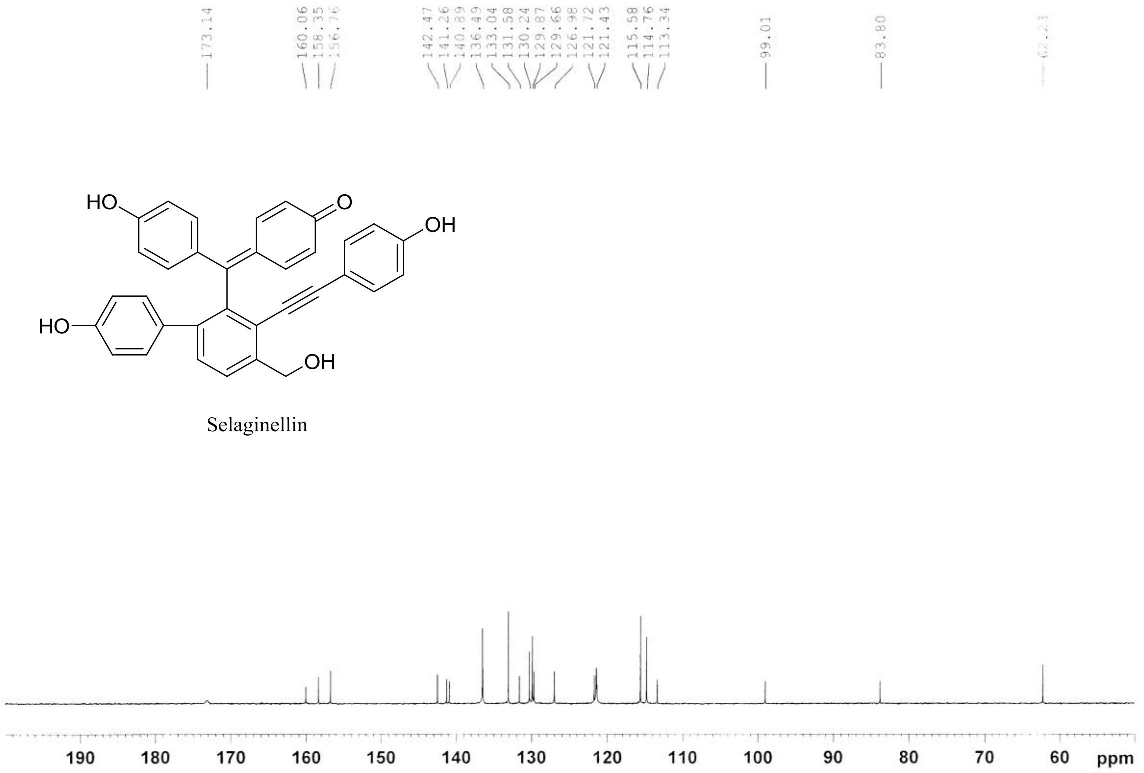

Figure S13: ^{1}H-^{1}H COSY of compound **3**

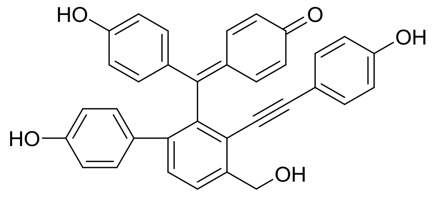

Selaginellin

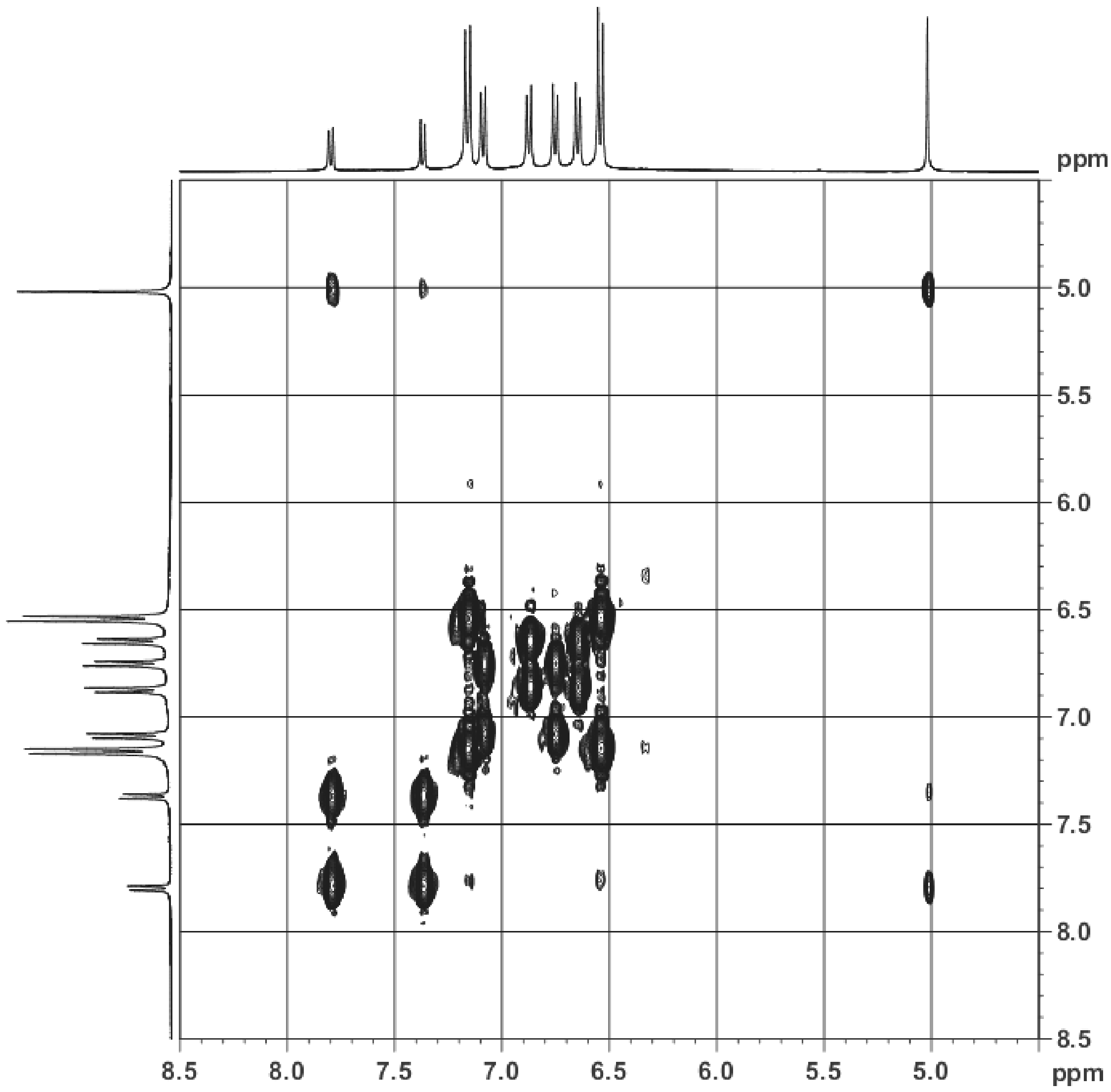

Figure S14: HMQC of compound **3**

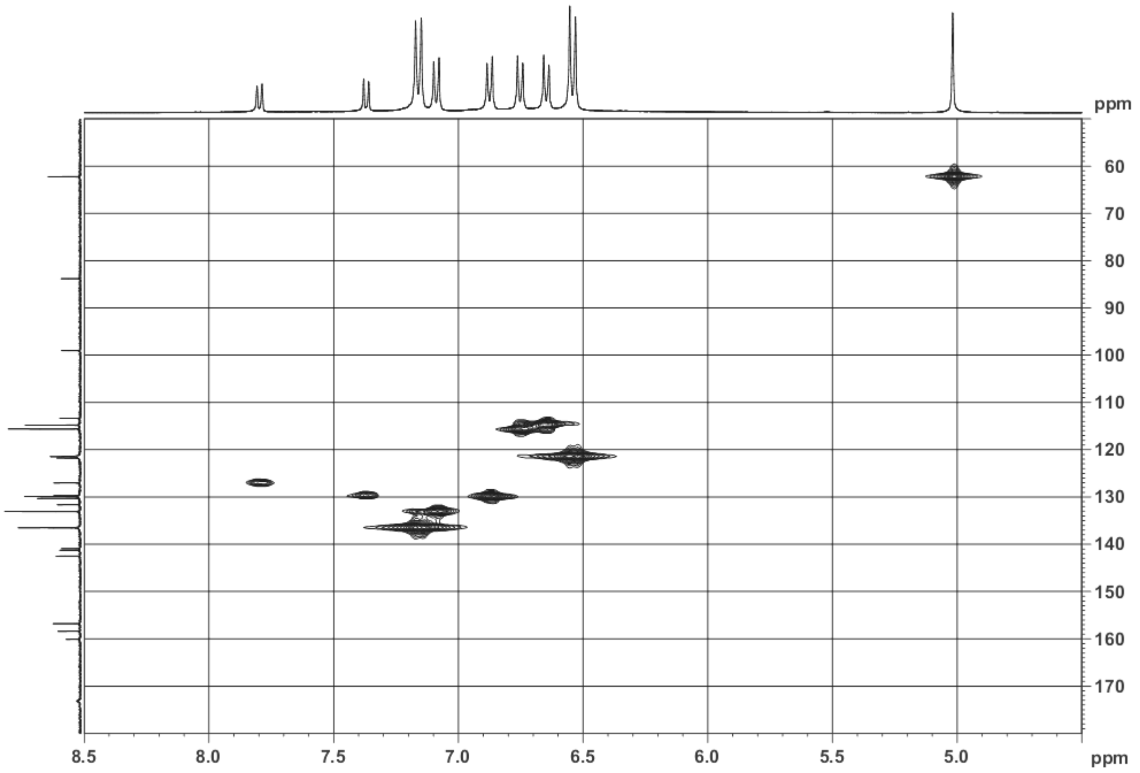

Figure S15: HMBC of compound **3**

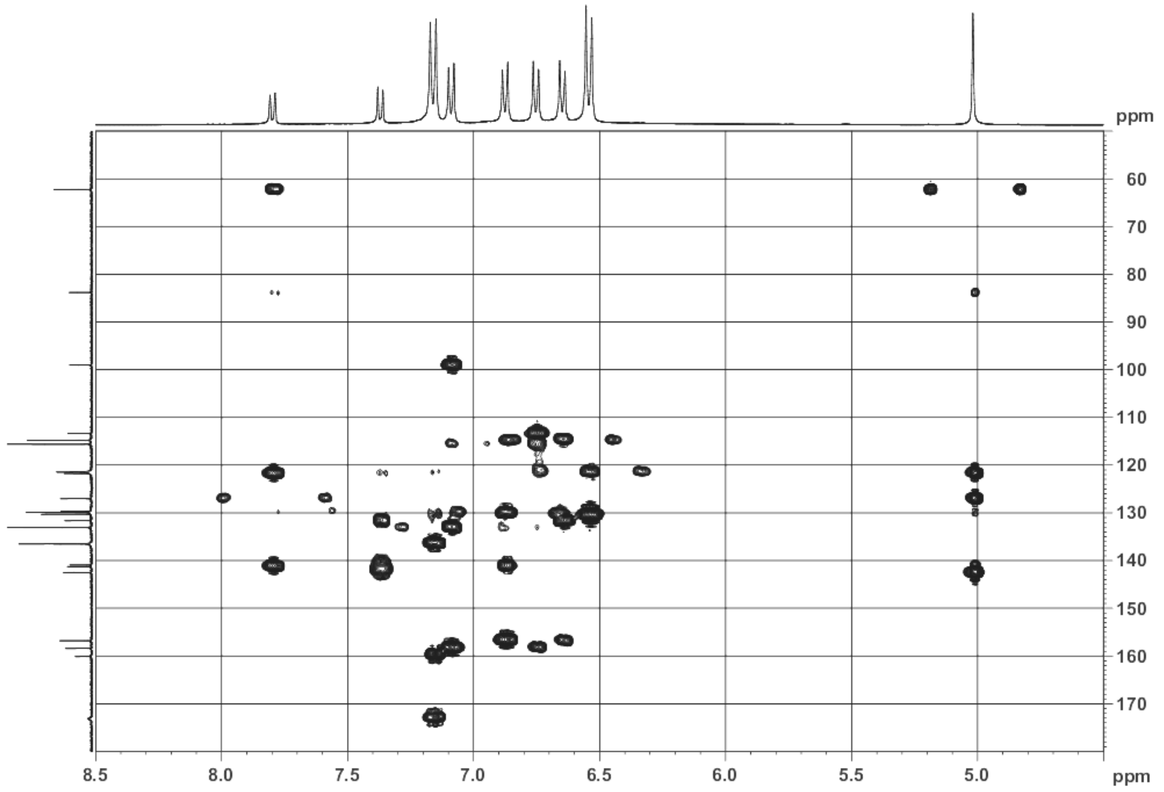

Figure S16: ^{1}H NMR (400 MHz, CD_3COCD_3) of compound **4**

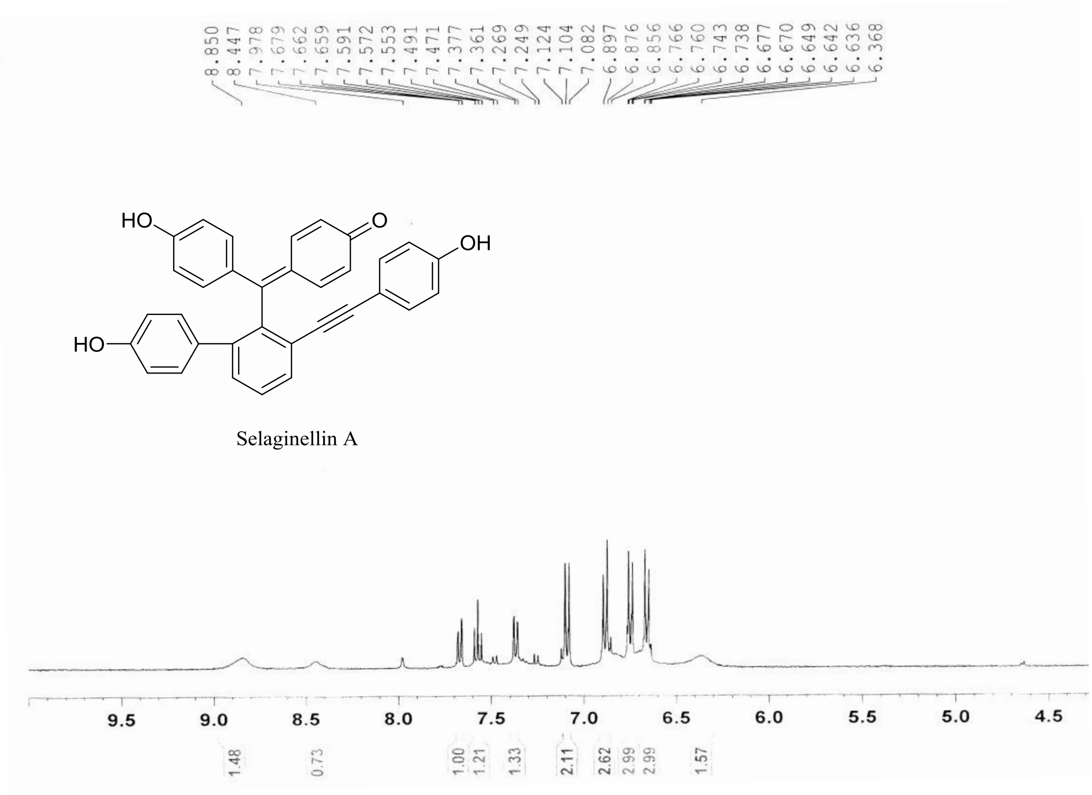

Figure S17: ^{13}C NMR (100 MHz, CD_3COCD_3) of compound **4**

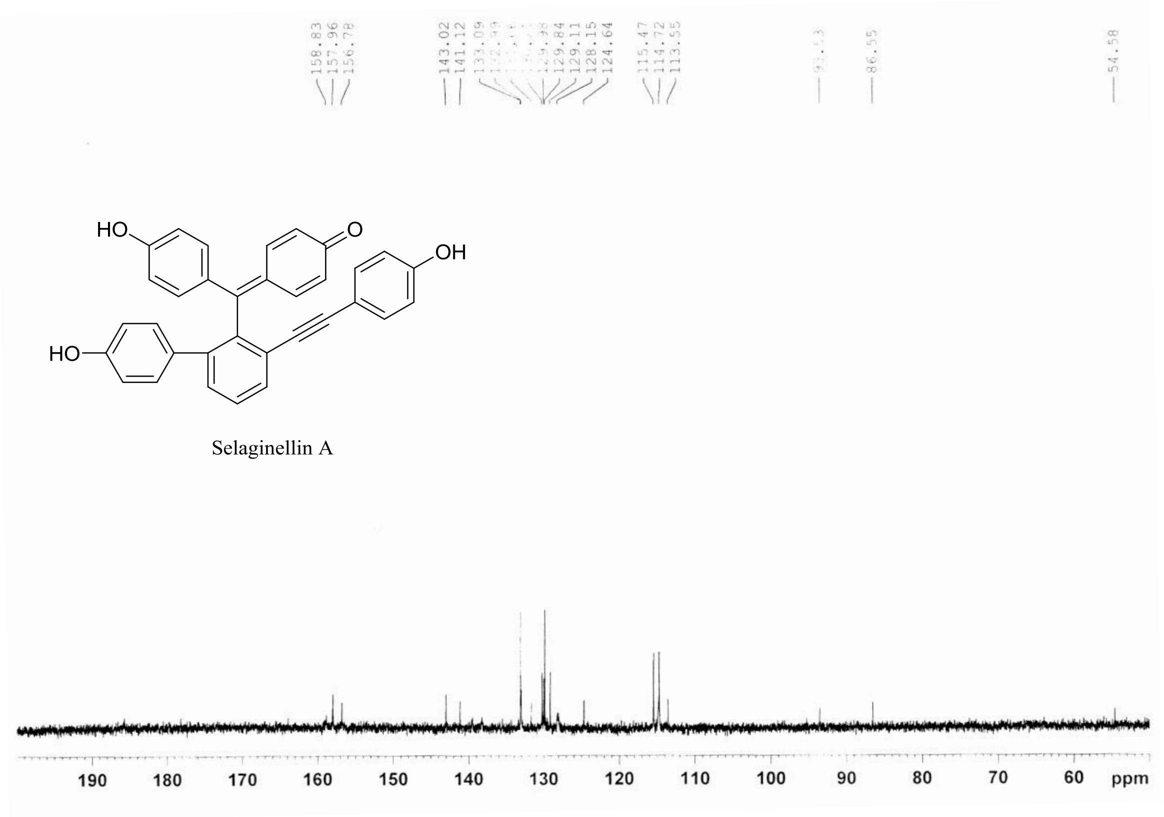

Figure S18: ^{1}H–^{1}H COSY of compound **4**

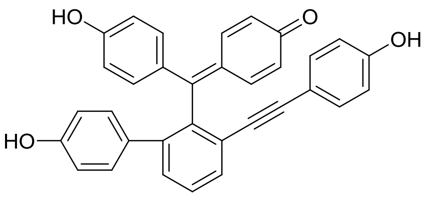

Selaginellin A

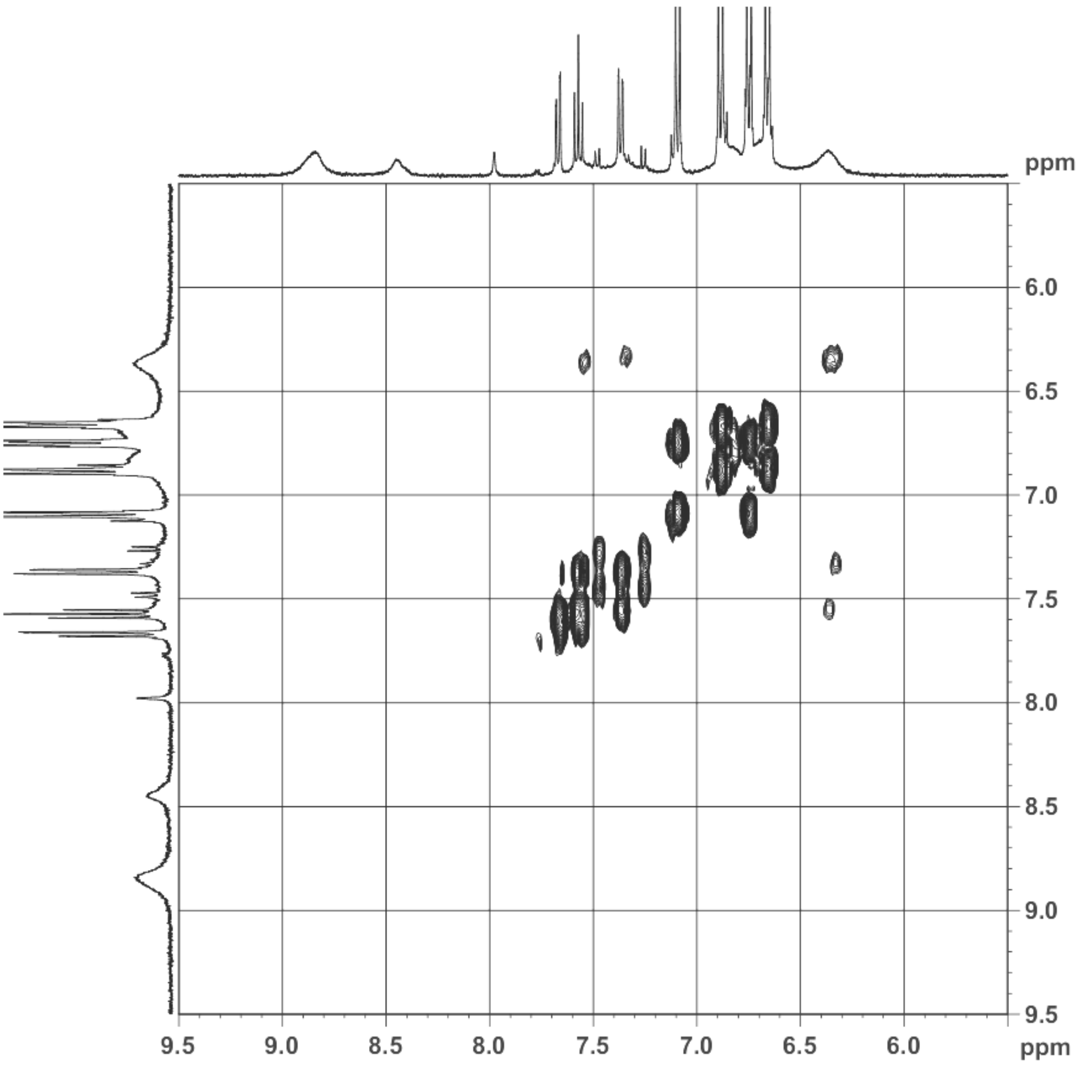

Figure S19: HMQC of compound **4**

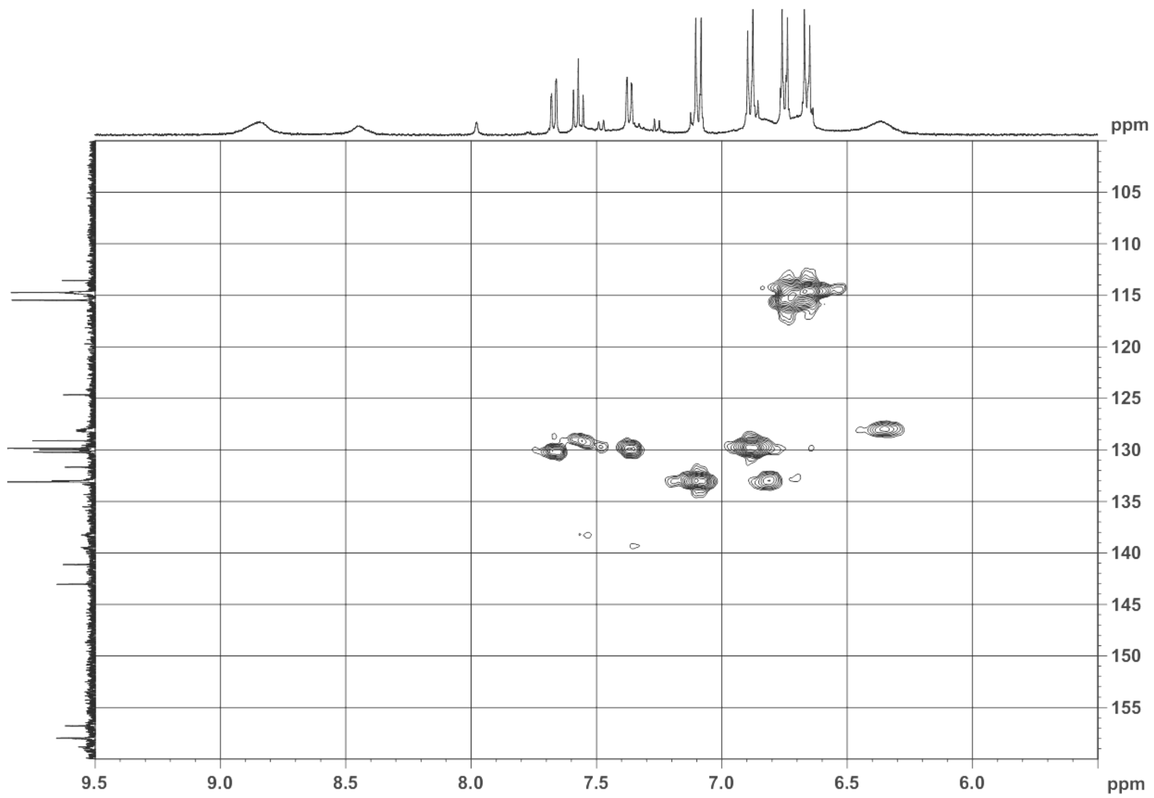

Figure S20: HMBC of compound **4**

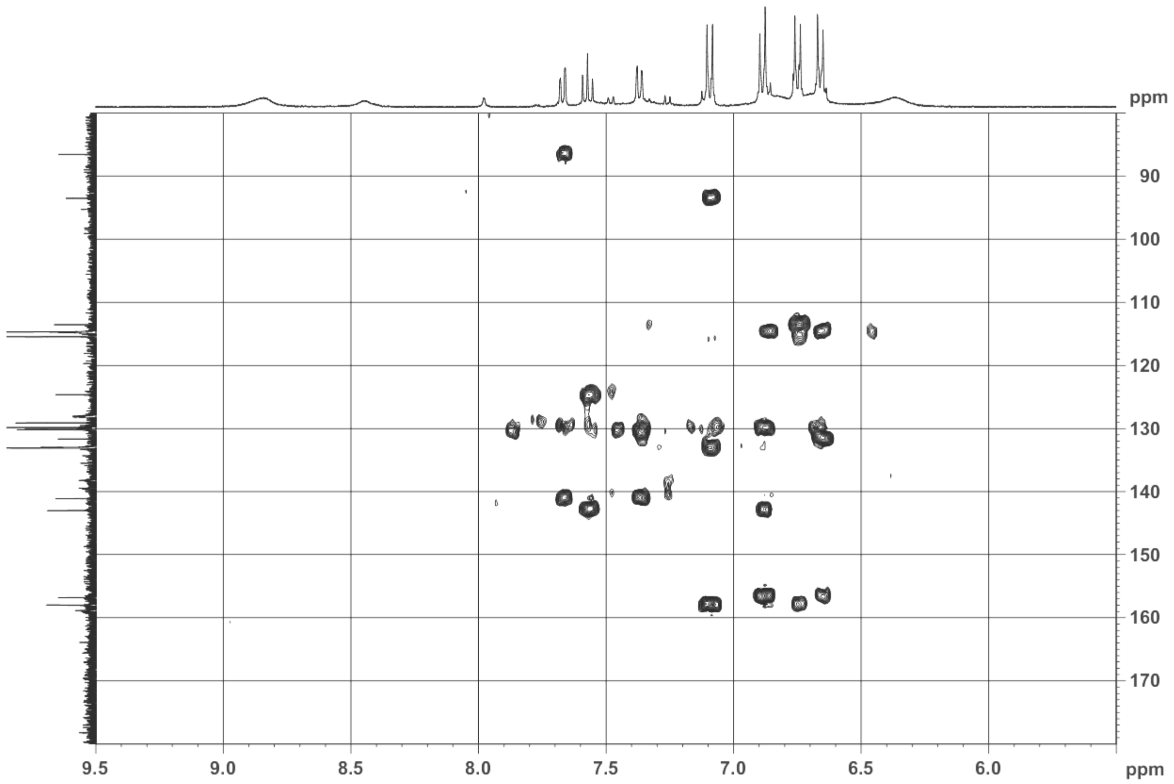

Table S1: Cytotoxic activity of compounds **1**–**3** evaluated with MTT assay.

Compounds	IC_{50} (μM ± SD)[a]
**1**	26.4 ± 0.80
**2**	28.5 ± 0.64
**3**	33.1 ± 1.21
Cisplatin[b]	15.4 ± 1.10

[a]The values represent means ± SD of three independent experiments

[b]Positive control

Table S2: Antioxidant activity of compounds **1**–**3** evaluated with ABTS and FRAP assays[a]

Compounds	ABTS assay	FRAP assay
	IC_{50} (μM ± SD)	Fe^{II} (μmol/μmol ± SD)
**1**	2.94 ± 0.014	1.10 ± 0.058
**2**	3.02 ± 0.029	1.05 ± 0.056
**3**	3.07 ± 0.012	0.94 ± 0.050
Trolox[b]	5.23 ± 0.019	0.87 ± 0.043

[a]Values are mean ± SD of three separated experiments.

[b]Reference compound.
